# Protein kinase CK2 localizes to sites of DNA double-strand break regulating the cellular response to DNA damage

**DOI:** 10.1186/1471-2199-13-7

**Published:** 2012-03-09

**Authors:** Birgitte B Olsen, Shih-Ya Wang, Tina H Svenstrup, Benjamin PC Chen, Barbara Guerra

**Affiliations:** 1 Department of Biochemistry and Molecular Biology, University of Southern Denmark, Odense, Denmark; 2 Department of Radiation Oncology, University of Texas Southwestern Medical Center, Dallas, TX, USA

## Abstract

**Background:**

The DNA-dependent protein kinase (DNA-PK) is a nuclear complex composed of a large catalytic subunit (DNA-PKcs) and a heterodimeric DNA-targeting subunit Ku. DNA-PK is a major component of the non-homologous end-joining (NHEJ) repair mechanism, which is activated in the presence of DNA double-strand breaks induced by ionizing radiation, reactive oxygen species and radiomimetic drugs. We have recently reported that down-regulation of protein kinase CK2 by siRNA interference results in enhanced cell death specifically in DNA-PKcs-proficient human glioblastoma cells, and this event is accompanied by decreased autophosphorylation of DNA-PKcs at S2056 and delayed repair of DNA double-strand breaks.

**Results:**

In the present study, we show that CK2 co-localizes with phosphorylated histone H2AX to sites of DNA damage and while CK2 gene knockdown is associated with delayed DNA damage repair, its overexpression accelerates this process. We report for the first time evidence that lack of CK2 destabilizes the interaction of DNA-PKcs with DNA and with Ku80 at sites of genetic lesions. Furthermore, we show that CK2 regulates the phosphorylation levels of DNA-PKcs only in response to direct induction of DNA double-strand breaks.

**Conclusions:**

Taken together, these results strongly indicate that CK2 plays a prominent role in NHEJ by facilitating and/or stabilizing the binding of DNA-PKcs and, possibly other repair proteins, to the DNA ends contributing to efficient DNA damage repair in mammalian cells.

## Background

A wide variety of lesion types can affect the DNA requiring the intervention of distinct and lesion-specific DNA-repair mechanisms. However, it is known that repair mechanisms may complement each other in some respects by sharing many protein components [1]. DNA double-strand breaks (DSBs) induced, for instance, by ionizing radiation (IR) and radiomimetic drugs are difficult to repair and extremely toxic although they do not occur as frequently as other types of DNA lesion [1,2]. DSBs are repaired by two main mechanisms: non-homologous end-joining (NHEJ) and homologous recombination (HR). NHEJ is the major repair mechanism in mammalian cells whereas HR is the predominant repair mechanism in budding yeast [2]. In NHEJ, DNA lesions are recognized by the Ku70/80 heterodimer. Localization of Ku to sites of DSB serves to recruit other NHEJ proteins such as the catalytic subunit of the DNA-dependent protein kinase (DNA-PKcs), ligases and polymerases [3,4]. The convergence of so many proteins to sites of DNA lesion is thought to protect at first the DNA ends from nucleases attack and later to facilitate the repair process. DNA-PKcs is a Ser/Thr kinase characterized by a weak activity that is significantly enhanced in the presence of double-strand DNA and Ku [4]. Modulation of its activity is not exclusively regulated by the interaction with Ku on DNA ends. In this respect, it has been reported that DNA-PKcs undergoes autophosphorylation at multiple sites, which is followed by DNA-PKcs release from DSBs *in vivo *and loss of protein kinase activity *in vitro *[4]. The significance of these events is not fully understood, however, it has been suggested that autophosphorylation of DNA-PKcs favors a conformational change that may serve to facilitate subsequent repair steps by making the DNA ends more accessible for damage-responsive proteins to sites of DNA damage [5-7].

Protein kinase CK2 is an evolutionary highly conserved Ser/Thr kinase composed of two catalytic subunits α and/or α' and two regulatory β-subunits [8,9]. CK2 is often described as a tetrameric enzyme but evidence has suggested that the individual subunits do not exist exclusively within the tetrameric complex but also as free proteins [10-12]. CK2 is invariably elevated in tumors and highly proliferating tissues and the de-regulated expression of the catalytic subunits is causative of transformation especially in combination with the altered expression of oncogenes and tumor suppressor genes [13,14]. CK2 appears to be involved in a plethora of cellular processes including regulation of cell cycle progression, survival, proliferation and those associated with various diseases, particularly cancer, neurodegenerative- and inflammatory disorders [15,16]. Current data suggest that CK2 plays a role in DNA sensing and repair. CK2 has been shown to phosphorylate the scaffold protein XRCC1 thereby enabling the assembly and activity of DNA single-strand breaks repair at sites of chromosome breakage [17]. Moreover, this kinase has been reported to phosphorylate the N-terminal domain of the MDC1 adaptor protein enabling its interaction with the NBS1 subunit of MRN protein complex which is involved in the initial processing of DNA repair [18,19]. Recently, by employing DNA-PKcs-proficient glioblastoma cells, we have reported evidence that siRNA-mediated down-regulation of the CK2 catalytic subunits results in significant cell death, decreased DNA-PKcs autophosphorylation at S2056 and delayed DNA repair following induction of DSBs [20].

In this study, given the importance of DNA-PK in DSBs repair, we have further elucidated the role of CK2 with respect to the molecular mechanisms activated in response to DNA damage. We report for the first time evidence that CK2 co-localizes with phospho-histone H2AX (γ-H2AX) to sites of DSB. We show that CK2 associates exclusively with DNA-PKcs and that the binding increases upon treatment of cells with radiomimetic drugs. Furthermore, depletion of the CK2 catalytic subunits destabilizes the interaction of DNA-PKcs with Ku80, which indicates that the observed decreased DNA-PKcs autophosphorylation might be the consequence of lack of interaction between DNA-PKcs and DNA on sites of genetic lesions.

Taken together, the reported observations strongly suggest that CK2 is required for the NHEJ-dependent cellular response to DSBs for an efficient repair process by stabilizing NHEJ proteins at sites of DNA damage.

## Methods

### Cell culture

M059K, M059J, HCT116 and Cos-1 cell lines were cultured in Dulbecco's Modified Eagle's Medium (Invitrogen, Denmark) supplemented with 10% FBS (Biochrom AG, Germany). The H1299 cell line was cultured in Roswell Park Memorial Institute medium (Invitrogen) supplemented with 10% FBS. All cell lines were obtained from the American Type Culture Collection (Rockville, MD, USA) and maintained at 37°C under a 5% CO_2 _atmosphere.

### Cell transfection and treatments

Cells were transfected with a set of four small interfering RNA duplexes directed against CK2α and CK2α' (ON-TARGET plus SMARTpools, Dharmacon, CO, USA), respectively, using Dharmafect I transfection reagent (Dharmacon) for 72 hours according to the manufacturer's instructions. Where indicated, control experiments were performed employing cells treated with transfection reagent alone or in combination with scramble-siRNA (Dharmacon). DNA damage was induced by cell treatment with the radiomimetic drug NCS (0.5 μg/ml, a gift from Dr. Hiroshi Maeda, Kumamoto University, Japan) and 33 μM Cisplatin (Sigma, Denmark), respectively, or exposure to 20 J/m^2 ^UV irradiation (Stratalinker UV Crosslinker, Stratagene, CA, USA) and 10 Gy ionizing radiation (6 MV energy from an Elekta Synergy Accelerator, Elekta, Sweden), respectively. For transient overexpression of the indicated proteins, cells were electroporated with the corresponding expression plasmids by using the NEON™ Tranfection system (Invitrogen) according to the manufacturer's instructions.

### Plasmid constructs

The coding region of CK2α' cloned into pcDNA3.1-MycHis [21] was excised with HindIII/XhoI restriction enzymes and inserted into pDsRed-Monomer N1 or pEGFP-N1 (both from Clontech-Takara Bio Europe, France), digested with HindIII/SalI, for the expression of DsRed- and GFP-tagged CK2α', respectively. The following DNA-PKcs fragments: Fragment A (aa 515-830), Fragment B (aa 1079-1533), Fragment C (aa 2005-2555), Fragment D (aa 2768-3258) and Fragment E (aa 3414-4123) were all cloned into the mammalian expression vector pcDNA3.1-MycHis (Invitrogen) using reverse transcription PCR and total RNA extracted from HCT116 cells used as template. The following primer pairs were employed: for fragment A, forward primer 5'-CGGGATCCACCATGGGAAGAACTGGCAAATGGAAGGTGC-3' and reverse primer 5'-CGCTCGAGCACCACTTTATTAAATCCTTTCTGG-3'; for fragment B, forward primer 5'-CGGGATCCACCATGGGATCGCTTGCCTTTAATAATATCTACAG-3' and reverse primer 5'-CGCTCGAGCAGGAGAAGACTCACAAGGCG-3'; for fragment C, forward primer 5'-CGGGATCCACCATGGGAATCGAAATTAGGAAAGAAGCC-3' and reverse primer 5'-CGCTCGAGGGTATTTGAAGGTAACCTAG-3'; for fragment D, forward primer 5'-CGGAATTCACCATGGGACAGGTCGTTCTGTACAG-3' and reverse primer 5'-CGCTCGAG TAGTTTCATAGCAAGTGAG-3'; for fragment E, forward primer 5'-CGGAATTCACCATGGGAACGCTGGCAGATTTCTGTG-3' and reverse primer 5'-CGCTCGAGTCCTTCCCAGGTTCTGCC-3'. PCR products were inserted into BamHI-XhoI (Fragments A, B and C) or EcoRI/XhoI (Fragments D and E) restriction sites. The correctness of the sequences was verified by DNA sequencing analysis of the cloned fragments.

### Purification of recombinant proteins

Human recombinant CK2α was expressed and purified essentially as described [22].

### Clonogenic survival assay

M059K cells were transfected with siRNAs against CK2α or CK2α' and 48 hours after transfection, cells were incubated with 0.5 μg/ml NCS for additional 24 hours. Afterwards, cells were trypsinized and seeded in six-well plates and colonies were allowed to grow for 14 days. Subsequently, cells were washed once with PBS and stained with a solution containing 6% glutaraldehyde/0.5% crystal violet and de-stained with tap water. Colonies containing > 50 cells were quantified using the UVIdoc software (Uvitec Ltd., UK).

### Western blot analysis

Whole cell extracts and Western blots were as previously described [21,23]. The antibodies used were as follows: mouse monoclonal anti-CK2α (#218703, Calbiochem, UK); mouse monoclonal anti-β-actin (#A3853, Sigma); mouse monoclonal anti-DNA-PKcs, -Myc and -Ku80 (#sc-5282, #sc-40, #sc-33653, all from Santa Cruz Biotechnology, CA, USA); rabbit polyclonal anti-53BP1 (#4937, Cell Signaling Technology, MA, USA) and rabbit polyclonal anti-phospho-DNA-PKcs (p-S2056) (#18192, Abcam, UK). Rabbit polyclonal anti-CK2α' was obtained by immunizing rabbits with a specific peptide (SQPCADNAVLSSGTAAR) of human CK2α'. Rabbit polyclonal anti-phospho-DNA-PKcs (p-T2609) and -(p-T2647) were as described [23,24].

### Immunoprecipitation assay

Experiments were performed essentially as described previously [25] employing 1 mg cell lysate and monoclonal anti-Myc (#2278, Cell Signaling Technology), rabbit polyclonal anti-DNA-PKcs (#300-516A, Bethyl Laboratories, TX, USA) or anti-CK2α serum antibodies as indicated in the figure legends. Anti-CK2α serum was obtained by immunizing rabbits against the full-length protein. 500 μg crude extracts from cells expressing various DNA-PKcs deletion mutants and 0.5 μg human recombinant CK2α were employed in co-immunprecipitation experiments for mapping the DNA-PKcs interaction domains.

### Immunofluorescence and *in situ *PLA

M059K cells grown on cover slips were incubated with rabbit polyclonal anti-γ-H2AX (p-S139) antibody (#2577, Cell Signaling Technology, Denmark) followed by incubation with biotinylated swine anti-rabbit IgG (Dako, Denmark) and streptavidin-conjugated fluorescein-isothiocyanate (Dako) as previously described [21]. Cells were counterstained with 4',6-diamidino-2-phenylindole (DAPI) or Hoechst dye (Sigma), analyzed on a DMRBE microscope (400× magnification) equipped with a Leica DFC420C camera (Leica, Denmark) and processed using ImageJ software (NIH, MD, USA). Threshold adjustments were set to ensure quantification of true positively immunostained particles. For the analysis of γ-H2AX/CK2α' interaction by *in situ *PLA, cells grown on cover slips were incubated with mouse monoclonal anti-γ-H2AX (p-Ser139) antibody (#11174, Abcam) and a rabbit polyclonal antibody against CK2α'. Visualization of protein-protein interaction was performed following the manufacturer's instructions (Olink Biosciences, Sweden) and as reported earlier (20). Similarly, interaction between DNA-PKcs and Ku80 or histone H3 was analyzed labeling the cells with a mouse monoclonal antibody against DNA-PKcs and a rabbit polyclonal anti-Ku80 antibody (#sc-5282, #sc-9034, both from Santa Cruz Biotechnology) or a rabbit monoclonal anti-histone H3 (#4499, Cell Signaling Technology). Interactions between CK2α'/DNA-PKcs or CK2α'/Ku80 were analyzed using a rabbit polyclonal antibody against CK2α' and a mouse monoclonal anti-Ku80 or anti-DNA-PKcs antibodies (#sc-33653, #sc-9051, both from Santa Cruz Biotechnology). The number of *in situ *proximity ligation signals was determined using the freeware software BlobFinder http://www.cb.uu.se/~amin/BlobFinder.

### Statistical and densitometric analysis

The statistical significance of differences between means of two groups was evaluated by the two-tailed *t*-test (Student's *t*-test). The levels of significance are indicated in the figure legends.

## Results

### Down-regulation of CK2 significantly impairs cell survival and results in persistent phosphorylation of histone H2AX

**Figure 1:**
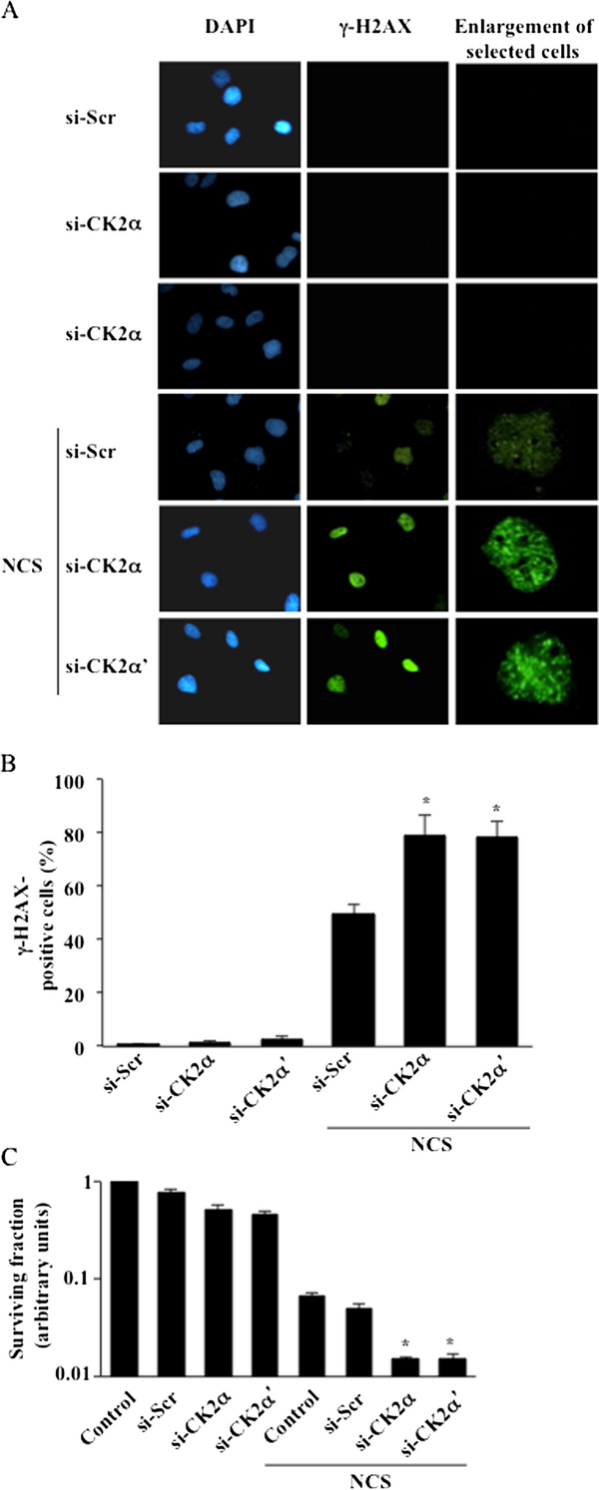
**Down-regulation of CK2 results in persistent γ-H2AX signal and reduced colony formation in human M059K glioblastoma cells**. **A**. Cells were transfected with scramble siRNA (si-Scr), CK2α or -α'-siRNA for 72 hours. Where indicated, 0.5 μg/ml neocarzinostatin (NCS) was added to the medium in the last 24 hours of incubation. Fixed cells were subsequently labeled with anti-γ-H2AX antibody and with a FITC-conjugated secondary antibody. Nuclei were visualized by DAPI staining. **B**. Quantification of γ-H2AX-positive cells was performed by using ImageJ software and expressed as percentage of the total number of cells in each sample. Bars indicate mean values +/- standard deviation (SD) from three independent experiments. *P < 0.0001 indicates statistically significant difference in the number of γ-H2AX-positive cells in CK2-depleted versus si-Scr-treated cells. **C**. Cells were transfected with CK2α-, -α'-siRNA or scramble siRNA for 72 hours. 0.5 μg/ml NCS was added in the last hour of incubation. Control, refers to cell treated with transfection reagent only. Cells were allowed to form clusters for 14 days. Colonies were visualized by staining with crystal violet as described in Experimental Procedures. Bar graph shows cell colonies quantification. Average values +/- SD from three independent experiments are shown relative to control (i.e. transfection reagent-treated) cells. *P < 0.005 denotes statistically significant difference in number of colonies formed as compared to si-Scr-tranfected and NCS treated cells.

In order to investigate the role of CK2 in the cellular response to DNA damage, human M059K glioblastoma cells were transfected with a pool of small interfering RNAs (siRNAs) targeting the individual CK2 catalytic subunits (i.e. si-CK2α and si-CK2α', respectively) or scramble-siRNA (si-Scr). Cells incubated in the absence or presence of 0.5 μg/ml neocarzinostatin (NCS) for 24 hours were analyzed by staining with anti-γ-H2AX antibody for the visualization of nuclear foci of DNA damage [26,27]. The analysis revealed higher γ-H2AX signal in CK2α- and -α'-knockdown cells (i.e. ~30%) with respect to cells treated with NCS alone or in combination with scramble-siRNA transfection (Figure 1A and 1B) indicative of impaired DNA damage signaling. A clonogenic survival assay was subsequently performed in order to test the survival ability of cells treated as indicated in Figure 1C. Cells incubated with NCS and transfected with scramble-siRNA displayed a 16-fold lower survival rate in comparison to cells transfected with scramble-siRNA alone. On the other hand, cells exposed to NCS and transfected with siRNA against CK2α or -α' showed a 3-fold lower survival rate with respect to cells transfected with scramble-siRNA and incubated with NCS. Overall, results indicate that CK2α- and CK2α'-knockdown cells display a similar DNA damage response characterized by persistent γ-H2AX signal and lower cell survival rate in comparison to cells transfected with scramble-siRNA and treated with NCS.

### CK2 is recruited to sites of DNA double-strand break

**Figure 2:**
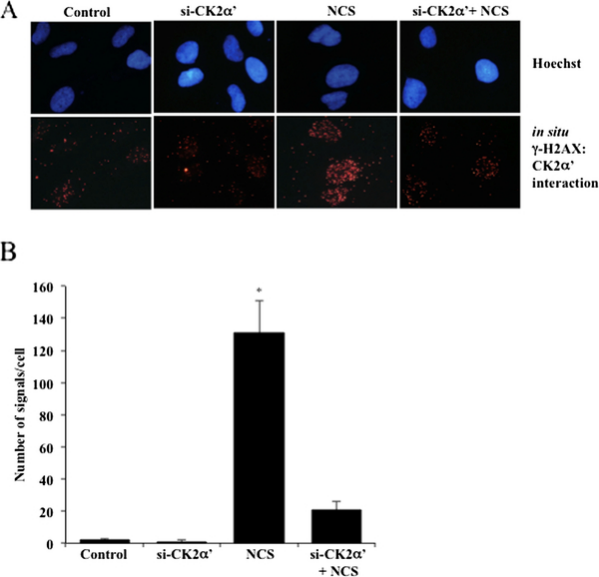
**CK2 co-localizes with γ-H2AX to sites of DNA damage**. **A**. Association between CK2α' and γ-H2AX was revealed by *in situ *PLA. Cells expressing or lacking CK2α' were left untreated or incubated with 0.5 μg/ml NCS for 1 hour before fixation and labeling with primary antibodies directed against the indicated proteins. Control, refers to cells transfected with si-CK2α' and stained with secondary antibodies. Cell nuclei were revealed by Hoechst staining. **B**. Quantification of the number of signals/cell as distinct fluorescent red spots was performed by computer-assisted image analysis as described in Experimental Procedures. Mean values +/- SD from three independent experiments are shown. *P < 0.0001 denotes statistically significant difference.

**Figure 3:**
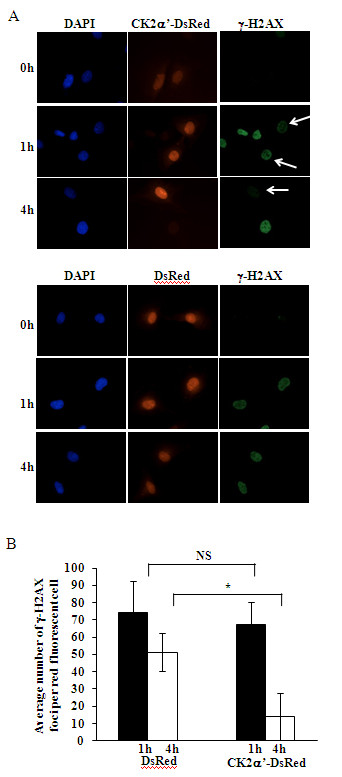
**Cells overexpressing CK2 exhibit a prompt response to induction of DNA double-strand break**. **A**. M059K cells transiently overexpressing CK2α'-DsRed or DsRed-fluorescent protein were incubated with 0.5 μg/ml NCS for the indicated times. Subsequently, cells were fixed and labeled with anti-γ-H2AX antibody. Cell nuclei were revealed by DAPI staining. Arrows: cells overexpressing CK2α'-DsRed are more efficient in DNA damage repair as compared to cells that do not overexpress the kinase. One representative experiment is shown. **B**. Average γ-H2AX foci number per red fluorescent cell as described in *A*. Mean values +/- SD from three independent experiments are shown. More than 100 cells have been analyzed per experiment. *P < 0.001 denotes statistically significant difference. NS, not significant.

As down-regulation of the individual CK2 catalytic subunits resulted in impaired DNA damage signaling, we investigated whether endogenous CK2 localizes to sites of DNA damage by looking at the association with γ-H2AX. We performed *in situ *proximity ligation assay (PLA), which enables the detection and quantification of protein-protein interactions or proteins in close proximity in native cells [28]. As shown in Figure 2, double staining of cells with antibodies directed against CK2α' and γ-H2AX, respectively, generated a significantly higher number of fluorescent signals (i.e. about 130-fold) in cells treated with NCS than in control- or CK2α'-depleted cells indicating that endogenous CK2 co-localizes with γ-H2AX to sites of DSB. To complement results described above, we examined whether the overexpression of CK2 affected DNA repair mechanisms induced by NCS treatment by looking at the dynamics of γ-H2AX in cells transiently expressing DsRed fluorescent protein (control experiment) or CK2α'-DsRed. As shown in Figure 3, an average of 74 γ-H2AX foci per cell were detected in cells expressing DsRed fluorescent protein 1 hour after addition of 0.5 μg/ml NCS to the incubation medium while cells expressing CK2α'-DsRed showed an average of 67 foci per cell under the same experimental conditions. After 4 hours from NCS treatment, 51 foci per cell were detected in DsRed-expressing cells while 14 foci per cell were still present in CK2α'-DsRed-expressing cells. This suggests that cells overexpressing CK2 display a prompt DNA damage response with respect to cells expressing endogenous levels of the kinase. We performed immunofluorescence-based experiments in order to clarify whether persistent phosphorylation of histone H2AX in cells depleted of CK2 and treated with NCS resulted from impaired γ-H2AX dephosphorylation or DNA repair defect. Cells were labeled with antibodies directed against p53 binding protein 1 (53BP1) which has been shown to play a key role in DNA damage repair [29]. Interestingly, we found that silencing of CK2 did not result in the persistence of 53BP1 foci as observed in cells expressing CK2 and exposed to NCS. These results suggest that persistent phosphorylation of histone H2AX may result from CK2-mediated DNA repair defect (Additional file 1: Figure S1).

### Down-regulation of CK2 regulates DNA-PKcs phosphorylation in response to DNA double-strand break induction

**Figure 4:**
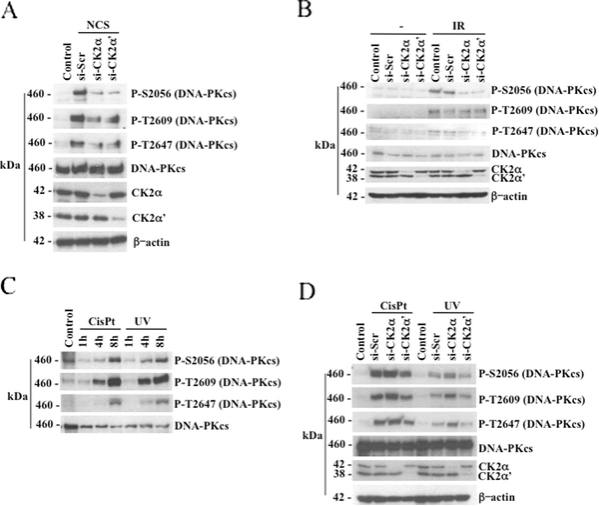
**CK2 depletion results in decreased DNA-PKcs phosphorylation in M059K cells following induction of DNA double-strand break**. **A**. Cells were treated as described in Figure 1. Whole cell extracts were employed for Western blot analysis of the indicated proteins. Detection of β-actin was used as loading control. **B**. Cells were treated essentially as described in Figure 1 except that DNA damage was induced by cell exposure to 10 Gy ionizing radiation. After 1 hour, cells were harvested and total cell lysates were prepared for Western blot analysis. **C**. M059K cells were treated with 33 μM cisplatin (cisPt) or exposed to 20 J/m^2 ^UV irradiation for the indicated time-points. Cell extract from control and treated cells was prepared and analyzed by Western blot employing antibodies against DNA-PKcs or its phosphorylated forms as indicated in the figure. **D**. Experiments were performed as described above and in Figure 1 except that cells were treated with cisPt (33 μM, 8 hours incubation) or exposed to UV (20 J/m^2^, 8 hours recovery) as indicated in the figure. Whole cell lysates were prepared for the analysis by Western blot of DNA-PKcs phosphorylation at S2056, T2609 and T2647. Experiments were performed at least three times obtaining similar results. Data from one representative experiment are shown.

Data reported above prompted us to investigate the mode by which CK2 contributes to DSBs repair mechanisms. We previously reported that down-regulation of the individual CK2 catalytic subunits causes a significant decrease in DNA-PKcs autophosphorylation at Ser2056 in response to NCS treatment [20]. Here, we extended the analysis of DNA-PKcs with respect to other phosphorylation sites which were reported to be rapidly phosphorylated *in vivo *upon exposure to ionizing radiation [30,31]. We obtained similar results with respect to the phosphorylation status of DNA-PKcs in cells transfected with scramble-siRNA or siRNAs against the individual CK2 catalytic subunits and incubated with either 0.5 μg/ml NCS for 1 hour (Figure 4A) or exposed to 10 Gy of irradiation (1 hour recovery, Figure 4B). The autophosphorylation of DNA-PKcs at Ser2056 decreased in CK2α- and -α'-knockdown cells, respectively, following induction of DSBs as compared to control cells where only DNA damage was induced. DNA-PKcs is phosphorylated at multiple sites within the so-called Thr2609 cluster that is essential for efficient DSBs repair [30]. As ATM (ataxia-telangiectasia mutated) has been reported to phosphorylate DNA-PKcs within the cluster [31], we looked at DNA-PKcs phosphorylation at two conserved sites, i.e. Thr2609 and Thr2647, respectively, that are likely to be relevant *in vivo *[3]. Western blot analysis showed a response similar to that observed in the case of Ser2056 phosphorylation in cells treated with NCS (Figure 4A) or exposed to IR (Figure 4B). However, the phosphorylation of Thr2609 did not decrease in CK2-depleted cells exposed to 10 Gy of irradiation (Figure 4B) as observed after NCS treatment (Figure 4A). Additional experiments were performed employing selective inhibitors of CK2 in order to address the question as to whether the observed CK2-mediated modulation of DNA-PKcs phosphorylation was dependent on CK2 kinase activity. Results obtained indicated that the kinase activity of CK2 does not regulate DNA-PKcs phosphorylation upon induction of DNA damage (results not shown). In addition to IR exposure, DNA-PKcs phosphorylation can be induced upon treatment with agents (e.g. hydroxyurea, camptothecin and cisplatin) or exposure to UV irradiation that all together cause DNA replication block [5]. M059K cells incubated with 33 μM cisplatin or exposed to 20 J/m^2 ^UV irradiation were subjected to a time-course experiment in order to determine the contribution of CK2 to the modulation of DNA-PKcs phosphorylation upon induction of replication-stress (Figure 4C). Phosphorylation of DNA-PKcs at Ser2056, Thr2609 and Thr2647 was markedly detected at 4-8 hours after UV irradiation or cisplatin treatment. We compared the phosphorylation status of DNA-PKcs in cells transfected with scramble-siRNA or siRNAs against the individual CK2 catalytic subunits and exposed to replication stress for 8 hours. As shown in Figure 4D, cellular depletion of CK2 did not significantly alter the phosphorylation of DNA-PKcs induced under replication stress. Interestingly, immunostaining of cells treated essentially as indicated above with anti-γ-H2AX did not result in changes in the number of detected foci in cells expressing and lacking CK2 expression, respectively, (Additional file 2: Figure S2).

**Figure 5:**
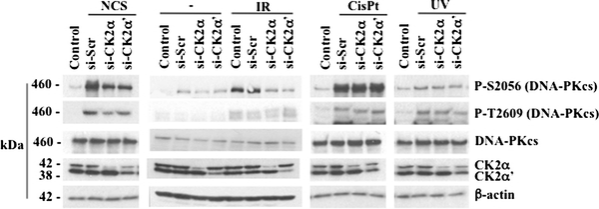
**Decreased phosphorylation of DNA-PKcs in CK2-depleted cells is not confined solely to glioblastoma cells**. The human non-small cell lung carcinoma H1299 cell line was subjected to treatments described in Figures 1 and 5. Western blot analysis of whole cell lysate was performed employing antibodies against proteins indicated in the figure. Experiments were performed three times obtaining similar results and data from one representative experiment are shown.

Next, in order to exclude the possibility that modulation of DNA-PKcs phosphorylation by CK2 knockdown expression was dependent on the particular cell line utilized in the experiments, we analyzed the phosphorylation status of DNA-PKcs in lysates from human H1299 non-small cell lung carcinoma cells. As shown in Figure 5, induction of DSBs by cell exposure to IR or treatment with NCS resulted in marked DNA-PKcs autophosphorylation at Ser2056 which was significantly reduced in CK2α- and -α'-depleted cells, respectively, as in the case of M059K glioblastoma cells. Phosphorylation of DNA-PKcs at Thr2609 decreased slightly in CK2-depleted cells exposed to NCS while no significant changes were observed in cells left untreated or exposed to IR. As observed in M059K cells exposed to UV irradiation or treated with cisplatin, depletion of CK2 in H1299 cells did not affect the phosphorylation levels of DNA-PKcs with respect to control experiments. Overall, results reported here indicate that the ability of CK2 to modulate the phosphorylation of DNA-PKcs occurs specifically in response to direct induction of DSBs and it is independent from the type of cells employed.

### Lack of CK2 destabilizes the association of DNA-PKcs with Ku80

**Figure 6:**
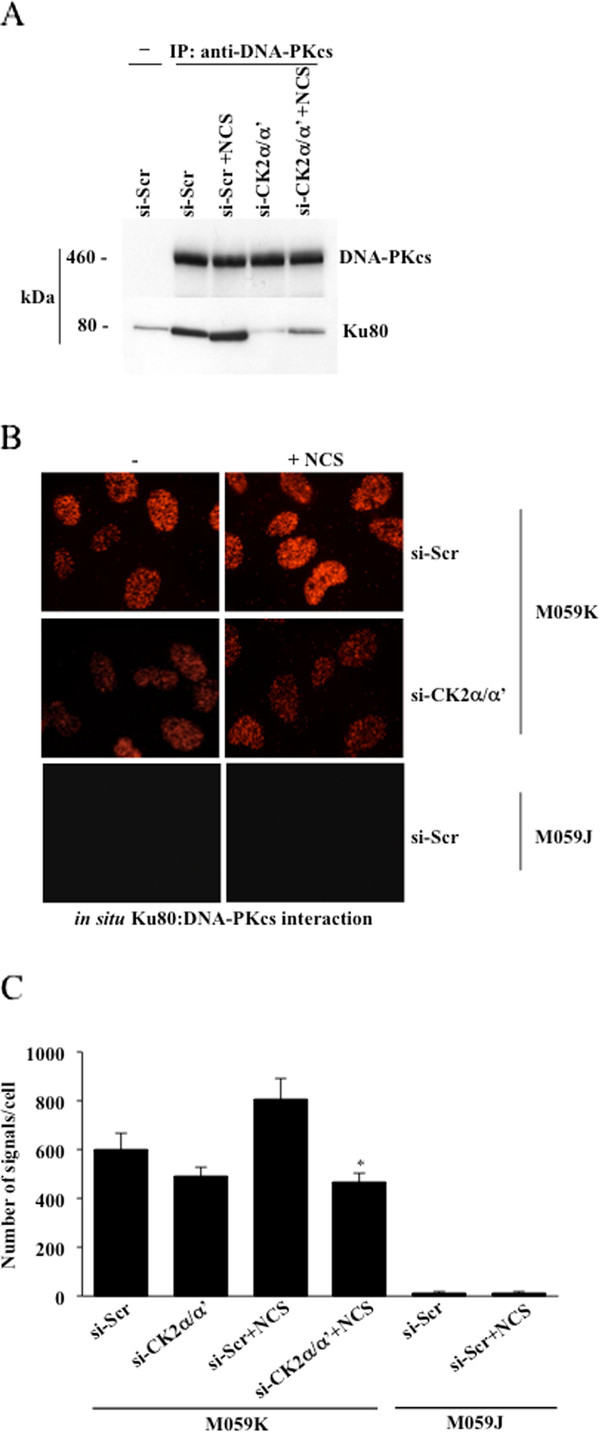
**siRNA-mediated knock-down of CK2 destabilizes the association between DNA-PKcs and Ku80**. **A**. Cells were transfected with si-Scr and si-CK2α/α', respectively. After 71 hours, cells were incubated with 0.5 μg/ml NCS for 1 additional hour as indicated in the figure. Whole cell lysate was subjected to co-immunoprecipitation (IP) with a rabbit polyclonal anti-DNA-PKcs antibody. Immunoprecipitated proteins were revealed by Western blot employing mouse monoclonal antibodies against the indicated proteins. A control experiment was performed where crude extract from cells transfected with scramble-siRNA was subjected to immunoprecipitation with normal rabbit serum (-). **B**. *In situ *association between DNA-PKcs and Ku80 was investigated in M059K- and DNA-PKcs-deficient M059J cells, treated according to conditions indicated in the figure, by *in situ *PLA. The molecular interaction is indicated by the presence of distinct red fluorescent spots in the cell nuclei. **C**. Quantification of the number of positive signals/cell was performed by computer-assisted image analysis. Mean values +/- SD from three independent experiments are shown. *P < 0.0001 denotes statistically significant difference between cell populations incubated with 0.5 μg/ml NCS for 1 hour and treated with si-Scr or siRNAs against CK2α and -α', respectively.

Down-regulation of CK2 leads to decreased DNA-PKcs autophosphorylation suggesting that CK2 either favors the interaction between DNA-PKcs molecules for their mutual phosphorylation *in trans *or mediates the association of DNA-PKcs with Ku in the presence of DNA. As Chen *et al.*, [5] demonstrated that the phosphorylation of DNA-PKcs at Ser2056 occurs directly between two kinase molecules, we hypothesized that the observed decreased autophosphorylation of DNA-PKcs in CK2-depleted cells was caused by defective recruitment of DNA-PKcs to sites of DNA damage. Therefore, we examined whether the interaction between DNA-PKcs and Ku80, whose expression levels do not vary in cells depleted of CK2 [20], was affected in CK2-siRNA-transfected cells. As shown in Figure 6A, immunoprecipitation experiments performed with an antibody directed against DNA-PKcs revealed a marked decrease in DNA-PKcs/Ku80 association in cells depleted of CK2α/α'. Similar results were obtained by performing *in situ *PLA (Figure 6B and 6C) where we looked at the association between DNA-PKcs and Ku80 directly in M059K cells treated or not with NCS. Here, we reported a ~50% decrease in the number of fluorescent signals in cells depleted of CK2 and exposed to NCS compared to control experiment where cells were transfected with scramble-siRNA and treated with NCS.

**Figure 7:**
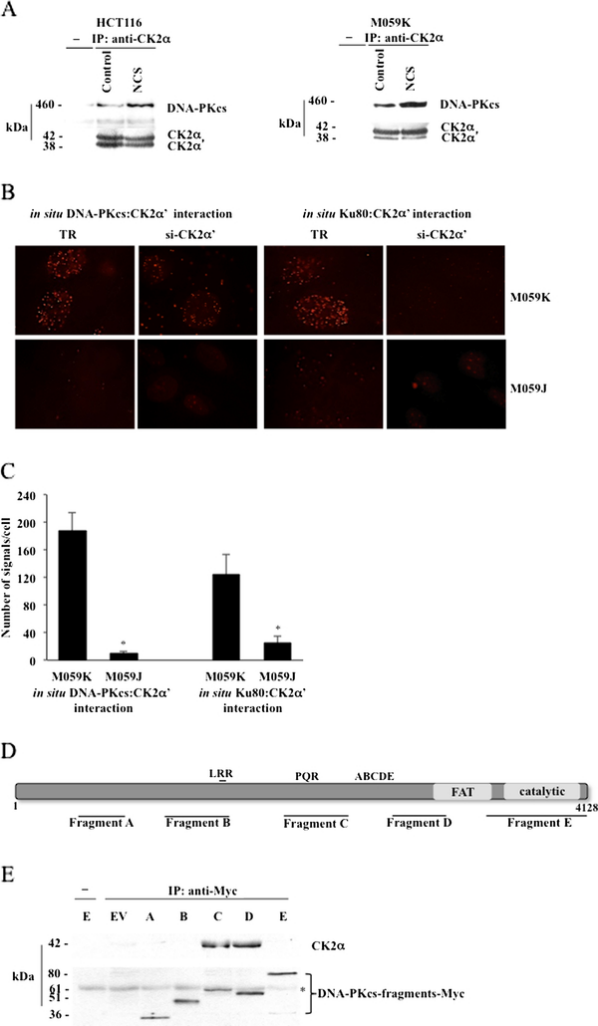
**DNA-PKcs associates with CK2. The complex formation is enhanced upon induction of DNA damage**. **A**. Cell lysates from HCT116 and M059K cells were employed in co-immunoprecipitation experiments in the presence of normal rabbit serum (-) or rabbit polyclonal anti-CK2α antibody. Immunoprecipitates were analyzed by Western blot with antibodies against the indicated proteins. **B**. The association between endogenous CK2α' and DNA-PKcs or Ku80 were explored by *in situ *PLA in M059K cells treated with 0.5 μg/ml NCS for 1 hour. Control experiments were performed employing either M059J- or M059K cells depleted of CK2α' as indicated in the figure. TR, transfection reagent. **C**. Quantification of number of positive signals/cell was performed as reported in Figure 2. Average values +/- SD from three independent experiments are shown. *P < 0.0001 denotes statistically significant differences between values obtained with M059K- and M059J cells, respectively, in the two assays. **D**. Schematic representation of DNA-PKcs linear sequence with some of the major domains indicated. LRR, leucine-rich region; PQR and ABCDE, clusters of phosphorylation sites; FAT, FRAP-ATM-TRRAP domain at the C-terminus; catalytic; PI3K- catalytic domain. The DNA-PKcs fragments employed in the study generated with a C-terminal Myc-tag are indicated below the bar. **E**. Whole extracts (500 μg) from Cos-1 cells expressing various DNA-PKcs deletion mutants (fragments A-E) were incubated with 0.5 μg purified human recombinant CK2α in the presence of either rabbit serum (-) or a rabbit monoclonal anti-Myc antibody. Precipitates were analyzed by Western blot employing mouse monoclonal antibodies directed against CK2α and Myc, respectively. EV indicates whole extract from cells transfected with empty vector and subjected to immunoprecipitation in the presence of recombinant CK2α (control experiment). *, denotes an unspecific protein band.

To address the question of whether CK2 interacts with DNA-PKcs and/or Ku80, we used whole lysates from HCT116 and M059K cells and performed co-immunoprecipitation experiments employing antibodies directed against CK2α. As shown in Figure 7A, CK2α/α' co-immunoprecipitated with endogenous DNA-PKcs in control cells and the association increased following induction of DSBs by NCS treatment. However, we did not detect any association with Ku80 protein (data not shown). Moreover, the analysis of cells left untreated or incubated with NCS did not show changes in the expression levels of both proteins (results not shown). In order to complement results obtained by co-immunoprecipitation experiments, we performed *in situ *PLA employing M059K and M059J cells and looked at the association between CK2α' and either DNA-PKcs or Ku80 in cells treated with NCS. As shown in Figure 7B and 7C, we confirmed the interaction between DNA-PKcs and CK2α' in M059K cells as previously reported [20]. As expected, a very low number of fluorescent signals was detected in M059J cells. Next, we looked at fluorescent signals deriving from *in situ *PLA performed employing antibodies directed against Ku80 and CK2α', respectively. Interestingly, the analysis of M059K cells revealed an average of ~110 positive signals per cell but this number was drastically reduced to ~20 positive signals per cell in M059J cells suggesting that CK2α' does not interact directly with Ku80. The evidence that CK2 interacts with DNA-PKcs, prompted us to define the DNA-PKcs-region(s) responsible for the interaction with CK2. Interaction studies were performed with human recombinant CK2α and a panel of Myc-tagged DNA-PKcs deletion fragments covering extensively the amino acid sequence of DNA-PKcs and transiently expressed in Cos-1 cells (Figure 7D and 7E). Experiments of co-immunoprecipitation performed employing anti-Myc antibody, revealed that two DNA-PKcs fragments, i.e. fragments C and -D comprising amino acid residues 2005-2555 and 2768-3258, respectively, interact specifically with CK2α (Figure 7E). Analysis of protein sequences preceding fragment A as Myc-fusions has not been possible due to difficulties in expressing N-terminal fragments.

## Discussion

NHEJ is the predominant repair pathway in mammalian cells that is activated in the presence of DSBs and requires the intervention of the DNA-PK complex for an adequate damage response. Although the importance of DNA-PKcs in DSB repair is well established and has been extensively studied, the precise mechanism by which this enzyme localizes to sites of DSB, phosphorylates itself and other repair proteins to promote NHEJ is still elusive. In this study, we report evidence that protein kinase CK2 contributes significantly to the DNA-PKcs-mediated cellular response to DNA damage. Lack of CK2 leads to marked cell death, persistent DNA damage and reduced survival rate in cells treated with radiomimetic drugs. We show that endogenous CK2 co-localizes with γ-H2AX to sites of DNA damage and its overexpression results in rapid decrease in the number of detected nuclear foci. This suggests that CK2 plays an important role in the DNA damage response. Experiments of co-immunoprecipitation and *in situ *PLA led to the observation that lack of CK2 destabilizes the Ku80/DNA-PKcs complex formation. These findings might explain the delayed DNA repair observed in cells lacking CK2 and, because survival of cells exposed to genotoxic stress is largely dependent on efficient repair of DSBs, the low clonogenic survival observed in CK2-depleted cells. The reported lack of association between Ku80 and DNA-PKcs in CK2-knockdown cells suggested that CK2 might mediate their interaction. However, we showed that endogenous CK2 binds to DNA-PKcs and the association is enhanced in response to DNA damage but we could not detect the presence of Ku80 in the complex formation. This has led to the hypothesis that CK2 interacts exclusively with DNA-PKcs facilitating and/or stabilizing the binding of the latter to DNA which has been reported to be weak [32]. In support of this notion, results obtained by *in situ *PLA where the binding of DNA-PK to chromatin components (i.e. histone H3) was tested in cells exposed to NCS, show that the association between DNA-PKcs and histone H3 is attenuated in cells lacking CK2 (Additional file 3: Figure S3). Data derived from the co-immunoprecipitation experiments and *in situ *PLA (Figure 7) suggest that CK2 binds exclusively DNA-PK. In this respect, the high level of fluorescent signal detected in M059K cells, which would indicate that CK2α' is also associated with Ku80, is almost completely suppressed in DNA-PKcs-deficient M059J cells. The association between CK2 and DNA-PKcs does not seem to be strictly dependent upon DSBs induction since we detected association also in the absence of DNA damage although to a lesser extent. This has been previously reported in interaction studies between mediator of DNA damage checkpoint protein 1 (MDC1) and NBS1 subunit of MRN comlex [18,19]. Here, the authors showed that the CK2-mediated phosphorylation of the STD-repeats of MDC1 enhances the binding to NBS1 thereby promoting the local concentration and/or stability of DNA damage regulators at sites of genetic lesions. MDC1 has also been shown to regulate DNA-PK autophosphorylation [33]. Our studies [20] indicate that it is not the catalytic activity of CK2 but rather the protein itself that regulates the interaction of DNA-PKcs with DNA at sites of damage underlining the multiplicity of the effects exerted by CK2. In light of the fact that CK2 might facilitate the binding of DNA-PKcs to DNA upon induction of DSBs, we believe that the apparent low phosphorylation levels of DNA-PKcs at S2056, T2609 and T2647 in CK2-depleted cells (Figures 4 and 5) might be caused by defective recruitment of DNA-PKcs to sites of DNA damage.

DNA-PKcs phosphorylation at T2609 in cells lacking CK2α or -α' and exposed to ionizing radiation did not lead to results similar to those obtained with cells treated with NCS (Figure 4). Currently, the reason for this discrepancy is not clear as both types of treatment generate DSB. However, T2609 is reported to be phosphorylated by all three members of the phosphoinositide 3-kinase-like family of protein kinases (PIKKs) and possibly other kinases [4], whose kinetic of phosphorylation might be differently regulated upon exposure of cells to IR or treatment with radiomimetic drugs. However, one cannot exclude that lack of chromatin remodeling may occur in CK2 deficient cells and lead to defective DNA-PK phosphorylation and other effects. In this respect, it has been shown that CK2 is implicated in chromatin remodeling by controlling the mobilization of HP1-beta through modulation of its phosphorylation at Thr51 [34].

Through experiments of mapping protein-protein interactions *in vitro *we defined two regions in DNA-PKcs responsible for the association with CK2α comprised between amino acid residues 2005-2555 (fragment C) and 2768-3258 (fragment D). It is conceivable that amino acids between these two regions which comprise the so-called ABCDE cluster of *in vivo *phosphorylation sites [35,36] are also involved in the interaction with CK2. It remains to be addressed whether the phosphorylation of these amino acids are affected upon binding of CK2 to DNA-PKcs. Fragment D comprises part of the so-called FAT domain, which is conserved in PIKKs and seems to regulate PIKKs kinase activity [37]. Part of the FAT domain comprises a high-affinity Ku binding (aa 3400-3420). Fragment E includes part of this Ku binding sequence. However, no interaction was found with CK2 suggesting that the association of DNA-PKcs with Ku and CK2, respectively, involves different domains.

## Conclusions

In summary, we show for the first time that protein kinase CK2 co-localizes with γ-H2AX to sites of genetic lesions and modulation of its expression levels affects the cellular DNA damage response. CK2 interacts with DNA-PKcs in mammalian cells in the absence of DNA damage and the association increases upon cell treatment with radiomimetic drugs. *In vitro *studies employing a panel of DNA-PKcs fragments and human recombinant CK2α show that the interaction is direct although one cannot exclude that the complex formation between CK2 and native, full length DNA-PKcs might occur in the presence of other DNA repair proteins and DNA. Evidence suggests that CK2 might facilitate and/or stabilize the binding of DNA-PKcs and possibly other proteins of NHEJ, to DNA ends contributing to efficient DNA damage repair. Additional experiments are necessary to further dissect the molecular mechanisms involved in NHEJ, nevertheless, the present findings point to CK2 as a prominent novel signaling kinase regulating DNA-PKcs fate in cells exposed to ionizing radiation or radiomimetic drugs.

## Competing interests

The authors declare that they have no competing interests.

## Authors' contributions

BBO carried out most of the experiments reported in the manuscript, participated in the design of the paper and critically revised it. S-YW carried out some experiments, BPCC provided reagents and critically revised the manuscript and THS carried out the molecular biology experiments. BG conceived the study, carried out some experiments and wrote the paper. All authors read and approved the final manuscript.

## Supplementary Material

Additional file 1 **Figure S1**. **53BP1 focus formation in cells treated with NCS**. Cells were transfected with scramble siRNA (si-Scr), CK2α or -α'-siRNA for 72 hours. Where indicated, 0.5 μg/ml neocarzinostatin (NCS) was added to the medium in the last 24 hours of incubation. Fixed cells were subsequently labeled with anti-53BP1 antibody and with a FITC-conjugated secondary antibody. Nuclei were visualized by DAPI staining. Click here for file

Additional file 2 **Figure S2**. **γ-H2AX focus formation in cells treated with cisPT or exposed to UV irradiation**. Cells were treated essentially as described in Figure 4D. After treatment, fixed cells were stained with anti-γ-H2AX and subsequently with a FITC-conjugated secondary antibody for revealing the presence of foci of DNA damage. Nuclei were visualized by DAPI staining. Click here for file

Additional file 3 **Figure S3**. ***in situ *** **PLA reveals interaction between DNA-PK and histone H3 in cells treated with NCS and expressing CK2**. **A**. Association between DNA-PKcs and histone H3 was investigated by *in situ *PLA in M059K cells treated as indicated in the figure and exposed to 0.5 μg/ml NCS for 1 hour. The molecular interaction is indicated by the presence of distinct red fluorescent spots in the cell nuclei. Control indicates cells treated with si-Scr and NCS and stained with the secondary antibodies after fixation. **B**. Quantification of the number of positive signals/cell was performed by computer-assisted image analysis. Mean values +/- SD from three independent experiments are shown. *P < 0.0001 denotes statistically significant difference between cell populations treated with si-Scr and siRNAs against CK2α', respectively. Click here for file
