# Supplementary figures and images for: Protein kinase CK2 localizes to sites of DNA double-strand break regulating the cellular response to DNA damage
Source: BMC Mol Biol. 2012 Mar 9;13:7. doi: 10.1186/1471-2199-13-7 (PMC3316135; doi:10.1186/1471-2199-13-7)

## Slide 1
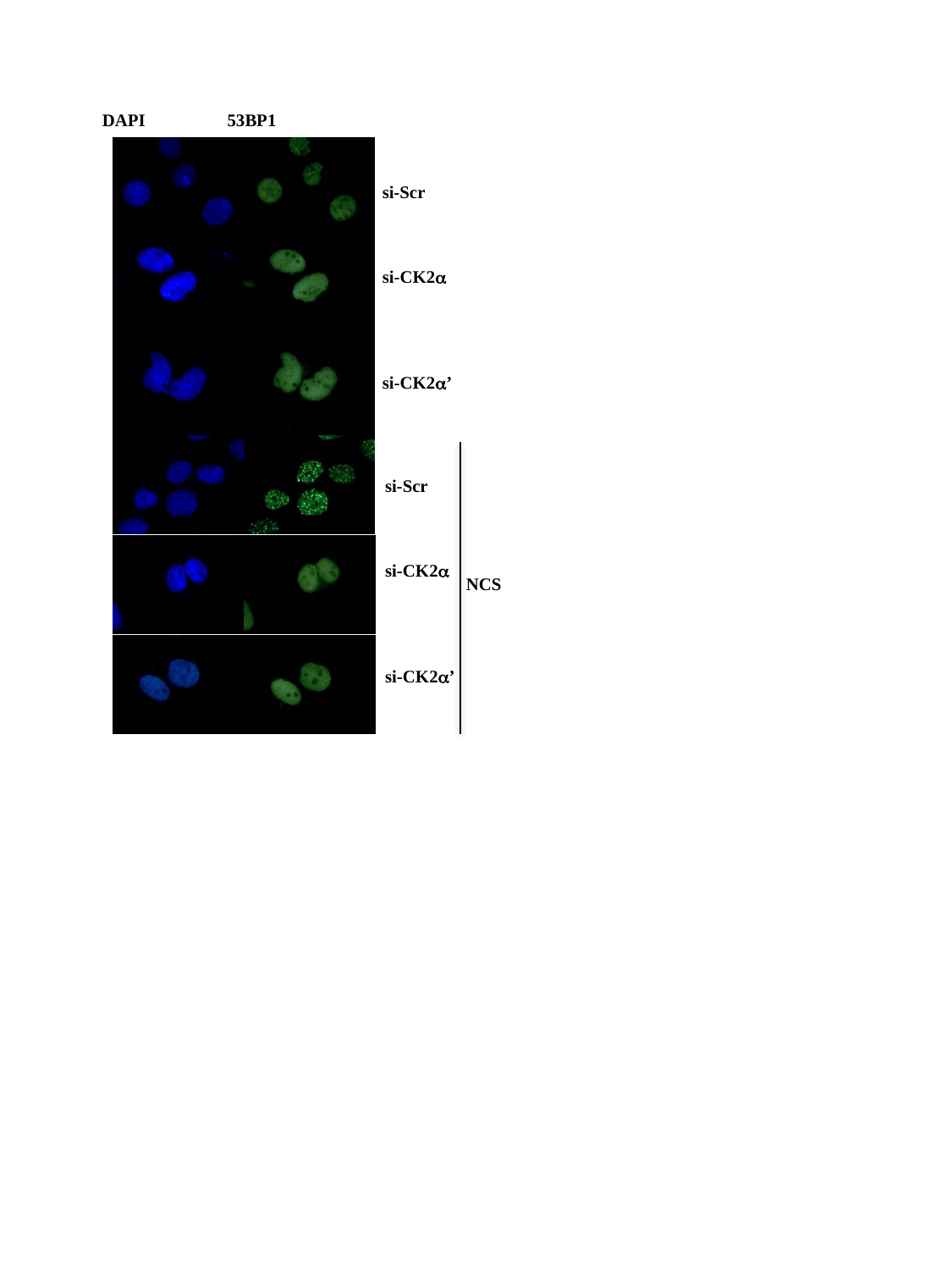

DAPI
53BP1
si-Scr
si-CK2a
si-CK2a’
si-Scr
si-CK2a
si-CK2a’
NCS

Supplement: Additional file 1 — Figure S1. 53BP1 focus formation in cells treated with NCS. Cells were transfected with scramble siRNA (si-Scr), CK2α or -α'-siRNA for 72 hours. Where indicated, 0.5 μg/ml neocarzinostatin (NCS) was added to the medium in the last 24 hours of incubation. Fixed cells were subsequently labeled with anti-53BP1 antibody and with a FITC-conjugated secondary antibody. Nuclei were visualized by DAPI staining. [file 1471-2199-13-7-S1.PPTX]

## Slide 1
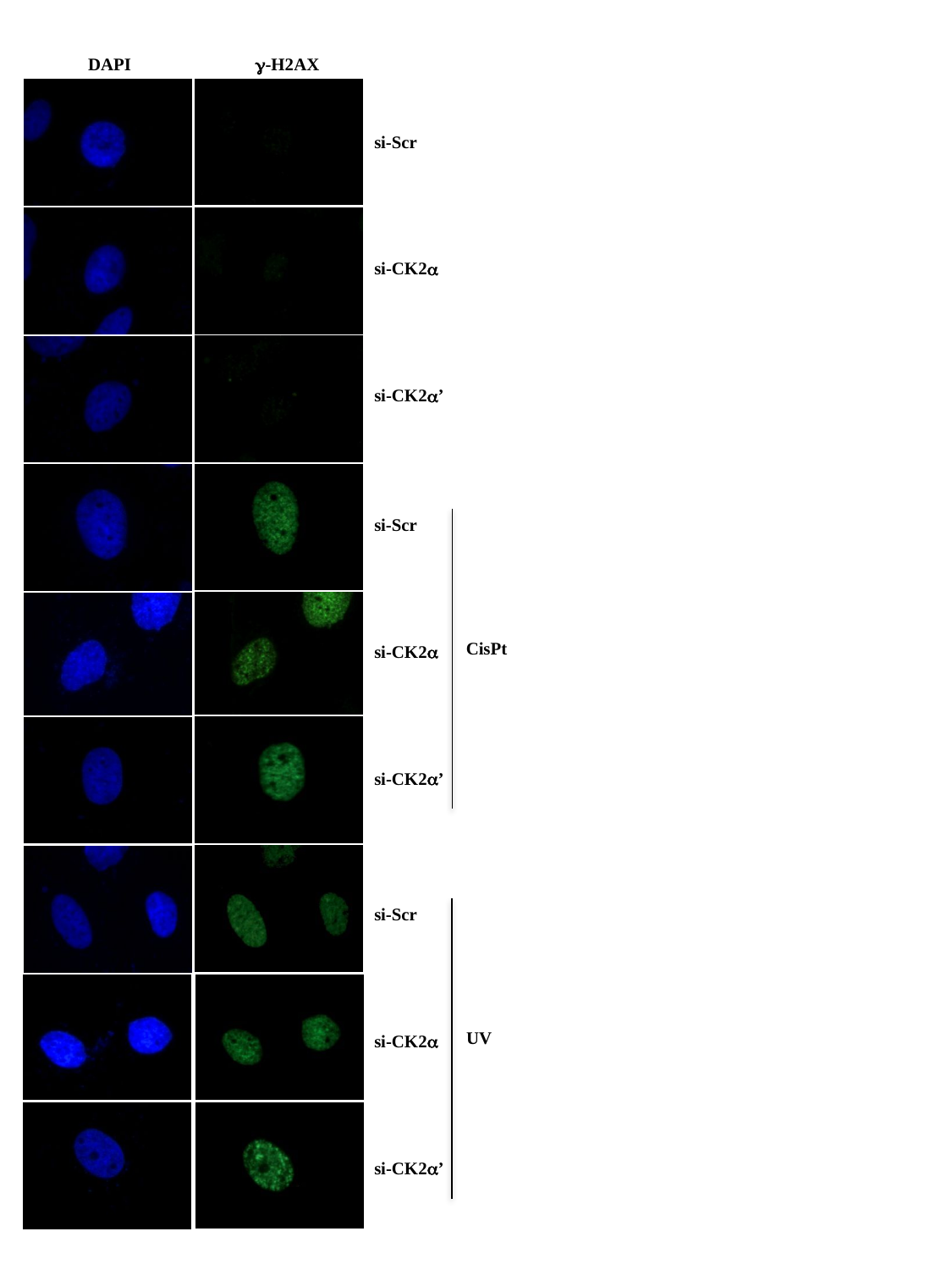

DAPI g-H2AX
si-Scr
si-CK2a
si-CK2a’
si-Scr
si-CK2a
si-CK2a’
CisPt
si-Scr
si-CK2a
si-CK2a’
UV

Supplement: Additional file 2 — Figure S2. γ-H2AX focus formation in cells treated with cisPT or exposed to UV irradiation. Cells were treated essentially as described in Figure 4D. After treatment, fixed cells were stained with anti-γ-H2AX and subsequently with a FITC-conjugated secondary antibody for revealing the presence of foci of DNA damage. Nuclei were visualized by DAPI staining. [file 1471-2199-13-7-S2.PPTX]
