# Supplementary material for: Protein kinase CK2 localizes to sites of DNA double-strand break regulating the cellular response to DNA damage
Source: BMC Mol Biol. 2012 Mar 9;13:7. doi: 10.1186/1471-2199-13-7 (PMC3316135; doi:10.1186/1471-2199-13-7)
Supplement: Additional file 3 — Figure S3. in situ PLA reveals interaction between DNA-PK and histone H3 in cells treated with NCS and expressing CK2. A. Association between DNA-PKcs and histone H3 was investigated by in situ PLA in M059K cells treated as indicated in the figure and exposed to 0.5 μg/ml NCS for 1 hour. The molecular interaction is indicated by the presence of distinct red fluorescent spots in the cell nuclei. Control indicates cells treated with si-Scr and NCS and stained with the secondary antibodies after fixation. B. Quantification of the number of positive signals/cell was performed by computer-assisted image analysis. Mean values +/- SD from three independent experiments are shown. *P < 0.0001 denotes statistically significant difference between cell populations treated with si-Scr and siRNAs against CK2α', respectively. [file 1471-2199-13-7-S3.PPTX]

## Slide 1
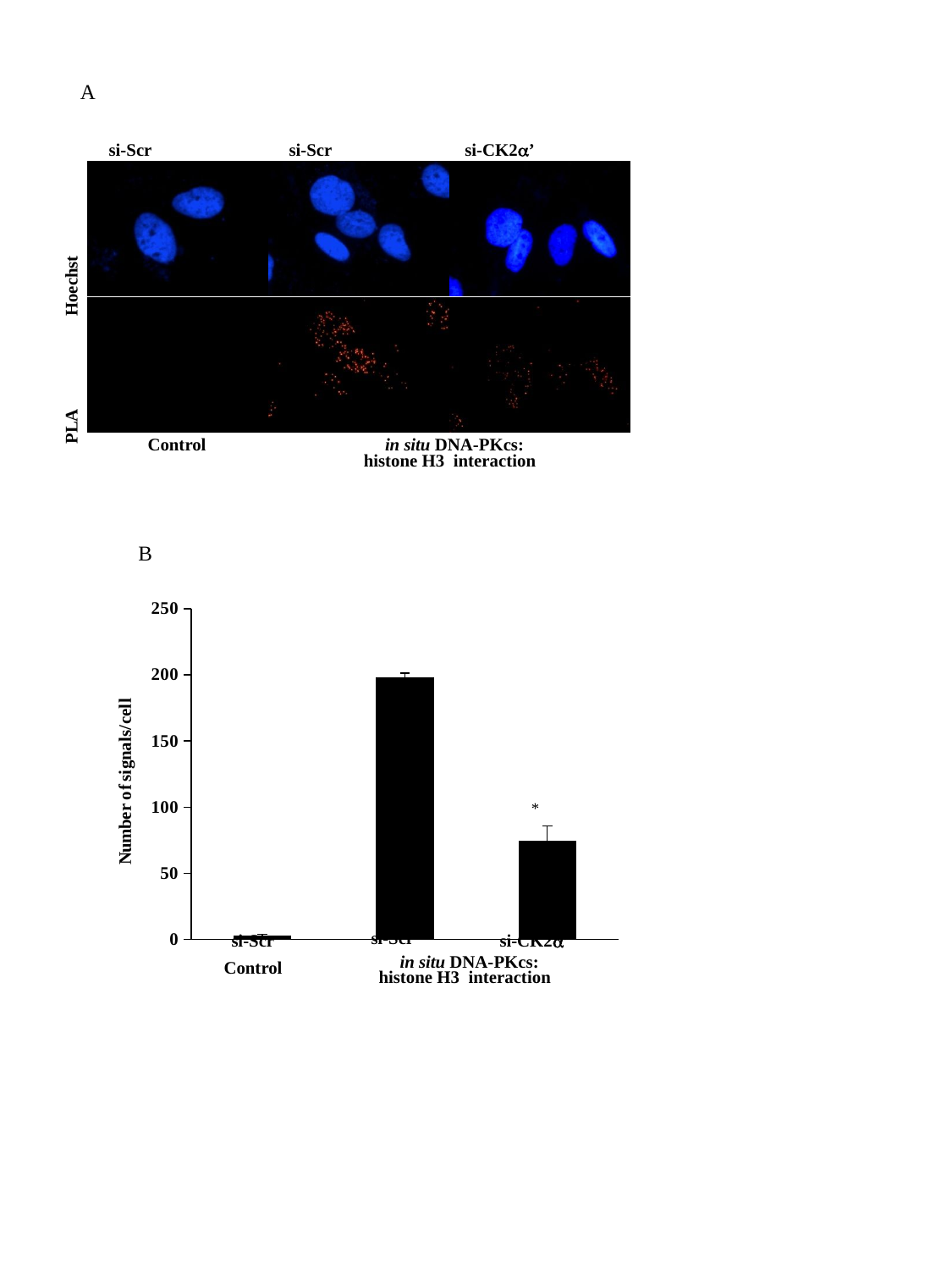

A
si-Scr si-Scr si-CK2a’
Hoechst
PLA
 Control
 in situ DNA-PKcs:
histone H3 interaction
B
### Chart
| Category | |
|---|---|
| Control | 2.76 |
| Scrambled | 197.95 |
| si-CK2 | 74.3 |*
si-Scr
si-Scr
si-CK2a’
 in situ DNA-PKcs:
histone H3 interaction
 Control
